# Chronic hepatitis E virus infection in a patient with leukemia and elevated transaminases: a case report

**DOI:** 10.1186/1752-1947-6-334

**Published:** 2012-10-02

**Authors:** Annika Gauss, Juergen J Wenzel, Christa Flechtenmacher, Mojdeh Heidary Navid, Christoph Eisenbach, Wolfgang Jilg, Wolfgang Stremmel, Paul Schnitzler

**Affiliations:** 1 Department of Internal Medicine IV, University of Heidelberg, Im Neuenheimer Feld 410, 69120, Heidelberg, Germany; 2 Institute of Medical Microbiology and Hygiene, University of Regensburg, Franz-Josef-Strauss-Allee 11, 93053, Regensburg, Germany; 3 Institute of Pathology, University of Heidelberg, Im Neuenheimer Feld 224, 69120, Heidelberg, Germany; 4 Department of Infectious Diseases, Virology, University of Heidelberg, Im Neuenheimer Feld 324, 69120, Heidelberg, Germany

**Keywords:** Hepatitis E virus, Chronic hepatitis, Autochthonous hepatitis E

## Abstract

**Introduction:**

Acute hepatitis E virus infection may cause mild, self-limiting hepatitis, either as epidemic outbreaks or sporadic cases, the latter of which have been reported in industrialized countries. Chronic infections are uncommon and have been reported in immunosuppressed patients, patients with human immunodeficiency virus infection, and patients with hematological malignancies.

**Case presentation:**

A 46-year-old Caucasian man was admitted to the gastroenterology clinic with a history of increasing transaminases, persistent exhaustion, and occasional right-side abdominal pain over the course of a 6-month period. B-cell chronic lymphocytic leukemia had been diagnosed several years earlier, and the patient was treated with rituximab, pentostatin, and cyclophosphamide. A diagnostic workup ruled out autoimmune and metabolic liver disease, hepatitis A-C, and herpes virus infection. A physical examination revealed enlarged axillary lymph nodes. The results of an abdominal ultrasound examination were otherwise unremarkable. Hepatitis E virus infection was diagnosed by detection of hepatitis E virus-specific antibodies. Blood samples were positive for hepatitis E virus ribonucleic acid with high viral loads for at least 8 months, demonstrating a rare chronic hepatitis E virus infection. Sequencing and phylogenetic analysis revealed hepatitis E virus genotype 3c with homologies to other European isolates from humans and swine, indicating an autochthonous infection.

**Conclusions:**

Usually, hepatitis E virus infection appears as an acute infection; rare chronic infections have been reported for transplant patients, patients with human immunodeficiency virus, and patients with hematological malignancies. The chronic nature of hepatitis E infection in our patient was most likely induced by the immunosuppressive B-cell chronic lymphocytic leukemia treatment. The differential diagnosis in patients with unexplained hepatitis should include hepatitis E virus infection, and appropriate laboratory analyses should be considered.

## Introduction

Signs and symptoms of hepatitis E virus (HEV) infection comprise jaundice, elevated transaminases, nausea, vomiting, myalgia, abdominal pain, and fever. In some cases, HEV infection may result in acute liver failure. The overall disease mortality is low, but fulminant hepatitis is frequent among pregnant women in HEV-endemic regions. Chronic hepatitis with persistent viral replication has been reported among transplant recipients infected shortly after transplantation [1,2]. Moreover, reports about chronic HEV infections in patients with concomitant human immunodeficiency virus (HIV) infection and patients with hematological malignancies have been published [3-5]. Epidemic outbreaks in developing countries are typically caused by HEV genotype 1 strains which are transmitted from human to human via the fecal-oral route [6]. In contrast, sporadic HEV cases in industrialized countries are most likely of zoonotic origin and usually are caused by HEV genotype 3 strains. Sporadic cases have been reported in France and other countries [7-9]. Only a few reports on zoonotic HEV infections in Germany exist [10]. Interestingly, recent reports demonstrate a surprisingly high HEV seroprevalence in German domestic pig stocks [11] and in humans with occupational exposure to pigs [12]. HEV-specific ribonucleic acid (RNA) was detected in porcine livers in southeastern Germany with high sequence homology to human HEV isolates [13]. Given these data, it is tempting to speculate that zoonotic and alimentary transmission routes play a role in sporadic human HEV cases, but the detailed mode of transmission is still not well understood. The subject of our report is a rare case of chronic HEV in an immunosuppressed patient with B-cell chronic lymphocytic leukemia (B-CLL).

## Case presentation

A 46-year-old Caucasian man who worked as a mechanic was transferred to the gastroenterology out-patient clinic by the department of hematology, where he was regularly treated for B-CLL. The reason for the visit was a continuous increase of transaminases over the previous 7 months (months 0 to 7). The patient reported persistent exhaustion and occasional right-side abdominal pain. B-CLL had been diagnosed 4 years earlier (initially Binet stage B; actual blood cell counts: leukocytes 13.6/nL, thrombocytes 224/nL, and hemoglobin 16.3g/dL). Owing to increasing night sweats and compression of the urinary bladder by lymphomas 2 years earlier (leukocyte count 61/nL), he was treated with rituximab, pentostatin, and cyclophosphamide for 3 months, resulting in partial remission. Maintenance therapy was performed by using rituximab and was finally abandoned in month 4 of increased transaminases because of severe fatigue and susceptibility to infections, after which the patient took no medication. At the time of referral to our department, the patient was in stable partial remission and B symptoms were absent. He had no history of recent travels to countries outside Germany, contact with animals, or consumption of venison, liver sausage, or offal. He had not received a blood transfusion prior to the infection.

**Figure 1:**
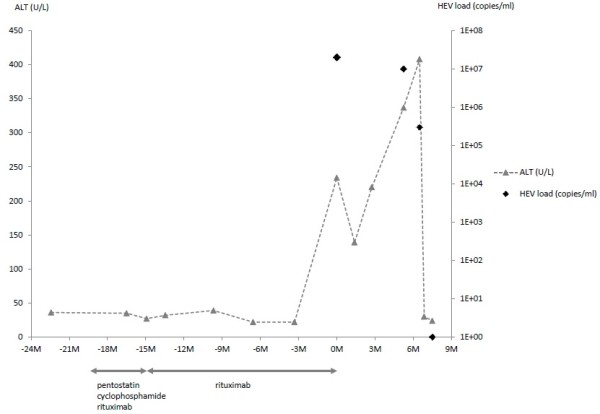
**Course of alanine transaminase and hepatitis E virus ribonucleic acid (HEV RNA) levels in the patient’s blood related to time indicated in months (M).** Intervals and types of chronic lymphocytic leukemia therapy are shown. The time scale on the x-axis is related to time point “0 M”, when elevated transaminase concentrations were measured for the first time. This was also the time point at which rituximab was administered for the last time. The diagnosis of HEV infection by positive HEV polymerase chain reaction was first made at 6.5 months; HEV RNA concentrations at 0 and 5.25 months were measured retrospectively. Unfortunately, no earlier samples were available to show when HEV RNA first appeared in the blood.

**Figure 2:**
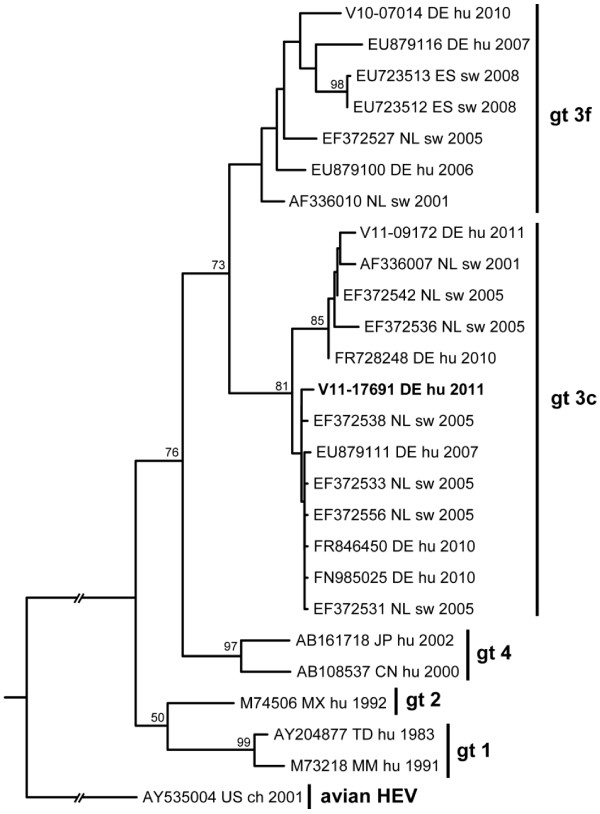
**Rooted maximum likelihood phylogenetic consensus tree for ORF1 nucleotide sequences of selected hepatitis E virus isolates.** The sequence of the presented case (V11-17691, bold) clusters in genotype 3 is shown. The selected sequences represent nearest homologs in GenBank and typical members of genotype 1, 2, and 4 [6]. An avian hepatitis E virus sequence was used as an outgroup. Numbers at the nodes indicate bootstrap values of greater than 50%. Sequences are denoted by GenBank ID, country, International Organization for Standardization country code, source, and year of isolation (or publication). ch, chicken isolate; CN, China; CZ, Czech Republic; DE, Germany; ES, Spain; FR, France; hu, human; GR, Greece; Gt, genotype; JP, Japan; MM, Myanmar; MN, Mongolia; MX, Mexico; NL, The Netherlands; SE, Sweden; sw, swine; TD, Chad; US, United States.

Normal alanine transaminase (ALT) was documented in our patient until 10 months prior to his admission to the gastroenterology department. No ALT results are available in the 3-month interval from 10 months until 7 months prior to his transfer to the department of gastroenterology. Therefore, there is a 3-month window during which the first increase of transaminases must have occurred. An elevated ALT concentration of 234U/L was first detected 7 months before referral to the department of gastroenterology (the month of referral is denoted as “month 0”). After the initial discovery of increased transaminases, moderate fluctuating transaminitis – month 1: aspartate transaminase (AST) 61U/L, ALT 139U/L; month 3: AST 108U/L, ALT 220U/L; month 5: AST 142U/L, ALT 337U/L; and month 6: AST 161U/L, ALT 408U/L – was observed. Bilirubin concentrations were repeatedly within the reference interval. A diagnostic workup ruled out autoimmune and metabolic liver disease, hepatitis A-C, and herpes virus infection. A physical examination revealed enlarged axillary lymph nodes. The results of an abdominal ultrasound examination were otherwise unremarkable. Finally, HEV was diagnosed in a blood sample by detection of anti-HEV antibodies and HEV RNA (month 7: 3.0×10^5^ copies/mL). Retrospectively, the presence of HEV RNA in serum was confirmed in two earlier samples (month 0: 2.0×10^7^ copies/mL and month 6: 1.0×10^7^ copies/mL). Samples from earlier visits were not available for analysis. Figure 1 shows ALT concentrations, HEV viral load, and phases of immunosuppressive therapy over time. Sequence determination and phylogenetic analysis revealed a novel HEV genotype 3 strain clustering with other European subgenotype 3c isolates (Figure 2).

Laboratory analyses ordered 10 days after referral to our department showed that transaminases had rapidly decreased to concentrations within the reference intervals without therapeutic intervention (Figure 1). Four weeks later (month 8), our patient was seen again in the department of hematology; transaminase concentrations were still normal, and HEV-RNA was not detectable from blood. Thus, it may be concluded that HEV infection was spontaneously cleared 7 to 8 months after the first referral. Lymphocyte counts in our patient’s blood were 0.8/nL at month 0, 0.7/nL at month 6, 9.4/nL at month 7, and 1.4/nL at month 8.

## Discussion

In this case report, our patient is a man from southern Germany with chronic autochthonous HEV infection. In industrialized countries, HEV is generally perceived as an imported disease, and travel to endemic regions is considered the primary risk factor. The disease is endemic in many countries in Asia and Africa and occurs both in sporadic forms and in waterborne outbreaks. However, recent evidence supporting the notion that autochthonous HEV infections are also widespread in many industrialized countries has accumulated. Phylogenetic analyses have shown that HEV genotype 1 is responsible for the majority of cases in developing countries, whereas the prevalent HEV genotype in Europe and North America is genotype 3 [10].

Our patient was referred to the department of gastroenterology with hepatitis of unknown origin. During a diagnostic workup, HEV was identified as the underlying disease. The diagnosis was based on three factors: first, a moderate fluctuating transaminitis with an ALT ranging from 61 to 408U/L, a typical pattern in chronic HEV infection [14]; second, the detection of specific HEV antibodies by enzyme-linked immunosorbent assay and immunoblot; and, third, the direct detection of virus-specific RNA by reverse transcription-quantitative polymerase chain reaction. Virus genotyping and subgenotyping were performed by sequencing and *in silico* phylogenetic analysis and revealed HEV subgenotype 3c. HEV RNA was detectable in three serum samples taken over an 8-month period. Thus, our patient tested positive for HEV RNA for more than 6 months, and this fits the definition of chronic HEV infection. In the course of an acute HEV infection, the highest level of viral nucleic acid in the blood is present before the occurrence of the first clinical symptoms and then rapidly declines in immunocompetent patients. After the onset of clinical symptoms, HEV RNA is usually detectable by polymerase chain reaction for only 14 to 28 days from blood, plasma, or serum. HEV genotype 3 has been reported for non-travel-associated infections in industrialized countries. Interestingly, a sequence database search revealed several closely related HEV sequences derived from swine as well as from humans in different European countries. In light of these findings and a recent report on the detection of HEV in porcine livers sold as food in southeastern Germany [3], our patient was questioned about his alimentary habits. However, ingestion of raw meat or offal and contact with animals (including pigs) were denied. Thus, the source of infection and mode of transmission remain unclear. Several reports in the literature suggest that patients under immunosuppression are at increased risk of acquiring autochthonous HEV infection with prolonged viral replication and potentially severe clinical consequences. Our patient had received therapy with anti-CD20 antibodies, pentostatin, and cyclophosphamide for his hematological malignancy. It is tempting to speculate that the chronic course of his HEV infection was, in fact, fueled by his therapeutically induced immunosuppression. The first step in treating patients with chronic infections is to reduce or stop immunosuppressive agents [14]. In our case, rituximab therapy was stopped because of severe fatigue and increased susceptibility to infections. Consequently, our patient’s lymphocyte count in the blood increased from 0.8/nL at the time of referral to 9.4/nL at month 7. This may be interpreted as an indicator of a simultaneous immune reconstitution and thus could explain the quick spontaneous clearance of the HEV infection and hepatitis between months 7 and 8.

Notably, severe complications have also been observed in patients with HEV infection after liver transplantation. In a recently published case, undetected HEV infection in a liver transplant donor actually caused chronic HEV and cirrhosis in the recipient [15]. In addition to acute and chronic HEV infections in patients with hematological malignancies [3,5], patients with HIV have been reported with chronic HEV [4].

In our patient, antiviral treatment was not initiated, because of the spontaneous recovery after immune reconstitution. However, beyond reduction of immunosuppressive therapy, treatment with ribavirin for at least 3 months seems to be the first treatment option for patients with chronic HEV, even though data are still limited [16].

## Conclusions

HEV is still regarded by many health-care professionals as a typical travel-associated disease. Consequently, HEV is often excluded from the differential diagnoses if a patient with acute hepatitis has no history of travel to known HEV-endemic regions. Thus, a considerable proportion of autochthonous infections presumably remain undiagnosed. In conclusion, patients with acute or chronic hepatitis of unknown cause during immunosuppression should be tested for HEV infection. Moreover, chronic courses of HEV must be considered in patients under immunosuppressive therapy.

## Consent

Written informed consent was obtained from the patient for publication of this case report and any accompanying images. A copy of the written consent is available for review by the Editor-in-Chief of this journal.

## Abbreviations

ALT: alanine transaminase; AST: aspartate transaminase; B-CLL: B-cell chronic lymphocytic leukemia; HEV: hepatitis E virus; HIV: human immunodeficiency virus; RNA: ribonucleic acid.

## Competing interests

The authors declare that they have no competing interests.

## Authors’ contributions

AG and PS helped to analyze and interpret the patient data regarding chronic HEV infection and helped to write the manuscript. CF, MHN, CE, WJ, and WS helped to analyze and interpret the patient data regarding chronic HEV infection. JJW helped to analyze and interpret the patient data regarding chronic HEV infection and performed polymerase chain reaction analysis. All authors read and approved the final manuscript.
